# Microplastics and Endocrine Disruptors in Typical Wastewater Treatment Plants in Megacity Shanghai

**DOI:** 10.3390/toxics12050345

**Published:** 2024-05-08

**Authors:** Yuxiao Tong, Manjun Xie, Hanwen Xv, Ruihua Sun, Qian Wang, Juanying Li

**Affiliations:** 1 College of Oceanography and Ecological Science, Shanghai Ocean University, Shanghai 201306, China; tyx0061@163.com (Y.T.); m210501173@st.shou.edu.cn (H.X.); jyli@shou.edu.cn (J.L.); 2 Shanghai Haibin Sewage Treatment Plant, Pudong, Shanghai 201306, China; xiemanjunkeynes@163.com; 3 Pudong Environmental Monitoring Station, Pudong, Shanghai 201306, China; rhsun2@hotmail.com

**Keywords:** microplastics, EDCs, WWTPs, environmental risk

## Abstract

The fast development of China’s urbanization has led to a notable release of emerging pollutants, including microplastics (MPs) and endocrine disruptors (EDCs). Generally, these pollutants enter the coastal environment through the discharge of wastewater treatment plants (WWTPs) and finally threaten the organisms in the receiving waterbody. The study investigated the environmental behavior of MPs and EDCs in two typical WWTPs in one of the megacities in China, Shanghai. The abundance of MPs in the influent ranged from 321 to 976 items/L. Four shapes (films, fragments, fibers, and microbead) were found, while fibers and films dominated. Transparent (31–63%) and white (20–47%) MPs were more frequently observed, while polyethylene terephthalate, cellulose, and cellophane were the main polymetric materials. The size of the MPs fell between 15.8 μm and 2220 μm, and the smaller one (<500 μm) dominated. The removal efficiencies of the two WWTPs for MPs ranged from 64% to 92%, and both WWTPs performed better for large pieces of MPs (>500 μm). For EDCs, total concentrations in the influent were detected, ranging from 113 to 2780 ng/L. Two groups, including phenolic estrogens (PEs) and steroid estrogens (SEs), were detected, and PEs, especially bisphenol A (BPA), were the predominant individuals among the studied EDCs. Specifically, PEs ranged from 82.8 to 2637 ng/L, while SEs ranged from 27.3 to 143 ng/L. The removal efficiencies of the WWTPs for EDCs varied (82.8–100%) as well, possibly due to the different treatment compartments and contamination load in the influent. Seasonal variations for both MPs and EDCs were observed. Specifically, concentrations of MPs and EDCs in WWTPs influent were higher in the wet season, as well as the removal efficiency. Furthermore, there was a correlation observed between the concentrations of MPs and EDCs, suggesting that MPs and EDCs may originate from the same source and that EDCs released by MPs cannot be ignored during treatment. Finally, the study evaluated the environmental risk of the effluents. MPs led to a minor risk (Level I), while EDCs might lead to an adverse impact on algae (RQs = 0.0014–0.024) and fish (RQs = 3.4–30.2). In summary, WWTPs received considerable amounts of MPs and EDCs. Although the WWTPs removed the contaminants efficiently, the environmental risk of the effluent needs to be noted.

## 1. Introduction

In recent decades, the urbanization of China has been fast, and Shanghai, as one of the most important megacities, uses and releases tons of emerging contaminants (ECs) from its human activities [1]. ECs included current-use pesticides (CUPs), pharmaceuticals and personal care products (PPCPs), endocrine disruptors (EDCs), microplastics (MPs), etc. These ECs are treated by sewage treatment plants, discharged into the coastal environment, and accumulate, eventually causing negative effects on ecosystems (biodiversity, sex, or age structure of the population) and aquatic life (individual death, developmental disorders, dysgenesis, etc.) [2]. However, the WWTPs are not designed for ECs specifically. Generally, primary, secondary, and tertiary treatment processes are included in WWTPs, and they are effective in eliminating pollutants such as organic substances and nutrients. The ECs could be eliminated via sedimentation, adsorption, and biodegradation [3,4]. However, the removal efficiencies are not stable, depending on the types, loads, and characteristics of the contamination. WWTPs have been identified as a non-negligible point source of ECs in the environment due to their unstable removal and continuous discharge [2].

Among the ECs, MPs and EDCs are receiving more and more concerns due to their rising adverse environmental impacts. Take MPs, for example; China produced more than 1.0 billion tons of plastic materials in 2020 (National Bureau of Statistics of China, 2000–2021). Many of the waste plastics are disposed of disorganized and broken into MPs (<5 mm). MPs in cities are partially discharged into WWTPs and finally released into the freshwater or coastal environment [5,6]. Thus, WWTP is an important point source of MPs. As reported, WWTPs in another megacity, Shenzhen in south China, produced 70.6–302 tons of MPs into its connected coast annually [7]. Based on the information provided by Ren, et al. [7], WWTPs across the country discharge 734–3100 tons of MPs into surface water each year, of which 220 to 950 tons are discharged annually into the marine environment. Thus, it is important to confirm and improve the removal of MPs in WWTPs. Although the WWTPs can achieve acceptable removal of MPs, the removal rate was unstable, and the removal efficiencies in different compartments were unclear [8]. For example, MPs could be removed by coagulation/flocculation (primary treatment) with a ratio of 47–82% and by A^2^O (secondary treatment) with a range of 47–82%, both of which fall within a wide range [9]. The removal rates highly depended on the contamination loads and composition patterns of MPs. Therefore, it is necessary to study the environmental behavior of MPs in and through the WWTPs in the megacity of Shanghai and evaluate the environmental risk of effluent that carries MPs.

Plastics and MPs have been confirmed as carriers for EDCs. In the polypropylene resin granules collected from the coastline in Japan, nonyl phenol (NP) was detected at a level of 0.13–16 ng/g [10]. During the aging process, plastics and MPs released a considerable amount of bisphenol A (BPA), and the aged plastics were identified as a continuous source of BPA [11]. Thus, the presence of MPs might be an indicator of the contamination of EDCs. It is necessary to study MPs and EDCs comprehensively to explain their environmental behavior. In addition to MP releases, industrial and medicinal procedures may potentially release EDCs as well. Specifically, steroid estrogens (SEs), such as estrone (E1), estradiol (E2), and estriol (E3), are used to regulate the development and reproduction function of human beings [12], while phenolic estrogens (PEs), such as BPA and NP, are important materials to produce surfactants, detergents, and paper products [13,14]. These EDCs were initially piped into the WWTPs and finally discharged into the receiving waterbody, e.g., the Yangtze River Estuary, in this study. The intensive discharge of EDCs and their metabolites into the environment might lead to hormonal dysfunction or reproductive abnormalities and perturb the stability of ecological systems [15]. However, as investigated by Xu, et al. [16], 12 EDCs were observed in the WWTP effluent in Hong Kong, in which the mean concentrations of E1 and NP were 5.25 ng/L and 4510 ng/L, respectively. Stasinakis, et al. [17] also reported a high level of BPA, which is of up to 5.76 μg/L. Similarly, the WWTPs are not specifically designed to eliminate EDCs, and the removal rates vary. The WWTPs in Hong Kong even reported elevated concentrations for some EDC individuals in their effluent [16]. Research on the removal of EDCs through WWTPs is essential for assessing the environmental risk of the effluent and improving treatment techniques, more specifically, for those in megacities that discharge significant amounts of ECs into the receiving water bodies.

Based on two typical WWTPs in Shanghai, the study evaluated the behavior and seasonal variation of MPs (sharp, color, polymers, and size) and EDCs (7 individuals) in and through different compartments in the WWTPs and confirmed removal efficiencies of different compartments. Then, the source of MPs and EDCs in the influent was identified. Subsequently, the relationship between the abundance of MPs and the concentration of EDCs was analyzed using principal component analysis (PCA). The environmental risk of MPs and EDCs in the effluent was calculated using the ecological risk index and risk quotient methods. Finally, the study could provide support for treatment optimization in WWTPs, as well as pollution management of ECs.

## 2. Materials and Methods

### 2.1. Materials and Chemicals

The chemicals for MP sample preparation included hydrogen peroxide (H_2_O_2_, analytical grade) and sodium chloride (NaCl, analytical grade). They were purchased from Sinopharm Group Chemical Reagent Co., Ltd. (Shanghai, China). For the analysis of EDCs (7 in total, Tables S3 and S4), their native standard solutions and surrogates (CRM) were needed. The standard substances, such as SEs (E1, E2, EE2, and E3), PEs (BPA and NP), and the required isotope-labeled surrogates were obtained from Dr. Ehrenstorfer GmbH (Augsburg, Germany). Solid-phase extraction (SPE) cartridges (Oasis HLB, 500 mg, 6 mL) to remove the interferences in the samples were provided by Waters Corporation (Milford, USA). The organic solvents (HPLC grade) were provided by Adamas (Shanghai, China), while the deionized water was produced by the Milli-Q^®^system in the laboratory (18.2 MΩ·cm).

### 2.2. Field Sampling

**Figure 1:**
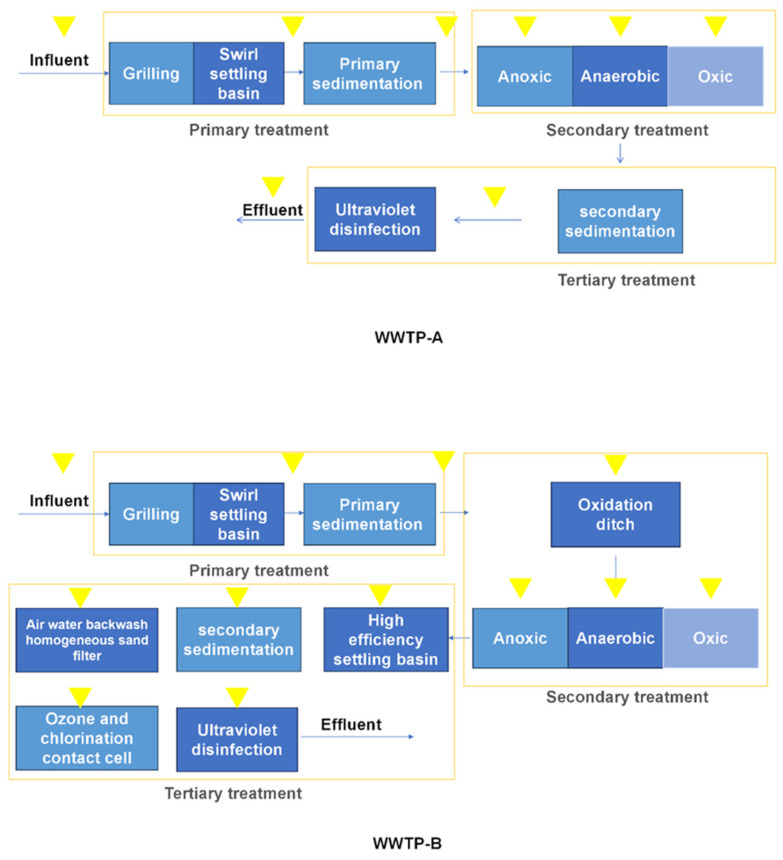
Treatment Compartments of WWTPs A and B. The yellow arrows represent sampling locations in the WWTPs.

The study focused on two typical wastewater plants in Shanghai, namely WWTP-A (2.8 million tons/day) and WWTP-B (400,000 tons/day). WWTP-A is the largest wastewater treatment facility in Shanghai, and WWTP-B includes advanced tertiary treatment processes, like ozone oxidation, which is not usual in traditional WWTPs. The main treatment processes of WWTP-A and WWTP-B are given in Figure 1. Water samples (n = 3, V = 1 L) were collected at the outlet of each treatment compartment in wet (July 2023) and dry (March 2023) seasons, respectively. Considering the hydraulic retention time (HRT) of the specific WWTP, each influent and effluent was collected and mixed every 24 h. Samples were kept in an amber bottle that had been rinsed with a solvent and pure water. Afterward, 4 M sulfuric acid (1/2000, *V*/*V*) was spiked to reduce biodegradation. The samples were transported to the laboratory, kept at 4 °C, and processed within 48 h.

### 2.3. Sample Preparation

Water samples were filtered through glass fiber filters (Whatman GF/F, 0.45 μm, Maidstone, UK). The suspended solid and MPs that remained on the filters were reserved for further treatment, and the filtrated water was processed with SPE cartridges for EDCs analysis.

The MPs that remained on the filters were washed by H_2_O_2_ into bakers, which were initially rinsed with pure water. Subsequently, the mixtures were placed into a shaker at a condition of 65 °C and 80 rpm for 72 h. Finally, the mixtures were processed again using membranes with a pore size of 5 μm before further analysis. More details have been provided by Su, et al. [18]

For EDCs, the filtrates were spiked with 0.2 g Na_2_EDTA and extracted by SPE with Oasis HLB cartridges (500 mg and 6 mL, Waters, Milford, MA, USA). The SPE cartridges were preconditioned with 10 mL methanol and 10 mL Milli-Q water. Then, water samples were loaded onto the cartridges at a flow rate of 10 mL/min. Afterward, the cartridges were rinsed with 10 mL of Milli-Q. The EDCs retained on the cartridges were eluted with 8 mL methanol and 8 mL methanol: Acetone (1:1, *v*/*v*), then the eluates were condensed to almost dry using a nitrogen evaporator and re-dissolved in 300 μL acetonitrile: water (3:7, *v*/*v*). The final extract was transferred to a 1 mL amber vial and stored at −18 °C until ultra-high-performance liquid chromatography-tandem mass spectrometry (UHPLC-MS/MS) analysis.

### 2.4. Instrumental Analysis

MPs were observed and shot using a stereo microscope and a photographic system (Nikon SMZ25, Nikon Corporation, Yokohama, Japan). Cooperating with the photographic system, the software (NIS-Elements D 4.50.00, Nikon Corporation, Yokohama, Japan) was used to record information about the physical properties of plastics, such as shape, color, and size. The imageJ (1.5) software was applied to label the scale of the photographic process. Based on the ratio of the pixel and scale, the size of MPs could be determined. Then, the polymeric component of MPs was analyzed using a Fourier transform infrared micro spectrometer (Nicolet iN 10 MX, Thermo Fisher, Waltham, MA, USA) with a transmission mode-MCT detector. More details of sample preparation for MPs could be found in the previous study [19].

Identification and quantification of EDCs were conducted using Ultra-High-Performance Liquid Chromatography-tandem mass spectrometry (UHPLC-MS/MS) equipped with an electrospray ionization source (Agilent 1290 Infinity UHPLC system and Agilent 6460 triple quadrupole MS; Agilent, Palo Alto, CA, USA) in multiple-reaction monitoring (MRM) mode. The analytes were separated on an Agilent Zorbax RR Eclipse Plus C18 column (95 Å pore size, 3.5 µm particle size, 2.1 mm inner diameter, and 150 mm length). The column temperature was set at 40 °C. The analysis EDCs was carried out using 0.1% ammonium hydroxide/H_2_O as eluent A and acetonitrile as eluent B at a flow rate of 0.3 mL/min; the injection volume was 10 μL. The ion source parameters for both positive (ESI+) and negative (ESI−) modes were selected as follows: gas temperature 300 °C, gas flow 7 L/min (antibiotics) and 10 L/min (EDCs), nebulizer 45 psi, sheath gas temperature 350 °C, sheath gas flow 11 L/min, and the capillary was 3500 V. Nitrogen gas was used as the collision gas. More details on elution gradients and precursors/products after MRM reaction are given in Tables S2 and S3.

### 2.5. Quality Control

To ensure the data quality of MPs, all laboratory testing is conducted in a specialized facility with restricted access. Blank samples are prepared using a blank filter with 1 L of pure water passing through. To minimize the contamination, all operators were asked to wear cotton lab coats to minimize the introduction of synthetic fibers. All the glass wares were rinsed with pure water. Blanks for EDCs were made for each of the ten samples. The MDL was estimated using a signal-to-noise ratio of 3 (Table S5). For the recovery study, water samples were spiked with standards at a concentration of 100 ng/L, and recovery ratios were also given in Table S5.

### 2.6. Risk Assessment

Environmental risk of MPs in the influent and effluent water was evaluated using the ecological risk index method, which was developed by Hakanson [20] and improved by Peng, et al. [21]. The calculations are given as follows:

(1) $$C_{f}^{i}=\frac{C^{i}}{C_{n}^{i}}$$

(2) $$T_{r}^{i}=\frac{P_{i}}{C^{i}}\times S_{i}$$

(3) $$E_{n}^{i}=T_{n}^{i}\times C_{f}^{i}$$

(4) $$RI=\sum_{i=1}^{n}E_{n}^{i}$$

$C_{f}^{i}$ is the pollution index which could be yielded using the measured abundance ($C^{i}$) divided by the criterion reference value ($C_{n}^{i}$), which in this case refers to the safe concentration of MPs in surface water (6650 items/m^3^) estimated by Everaert, et al. [22]. $T_{n}^{i}$ is the ecotoxicity response factor representing the toxicity and bio-sensitivity of the MPs. According to the study of Lithner, et al. [23], $P_{i}$ is the abundance of specific MP polymer (*i*), and $S_{i}$ is the hazard index of polymer (i) (Table S6). $E_{r}^{i}$ is the potential ecological risk index, which is determined based on $T_{r}^{i}$ and $C_{f}^{i}$ of each polymer. RI is the total ecological risk index of all types of MP polymers. As given in Table 1, ecological risk is classified as level I, II, III, and IV according to different RI values.

The ecological risk caused by EDCs was quantified based on the risk quotient method (RQ), which could be calculated by the measured concentration (MEC) divided by the predicted no-observable concentration (PNEC) [24]. The total RQ values (ƩRQ) of all the studied EDCs could be determined by summing up all the RQ values of individuals. If ƩRQ values are lower than 0.01, no risk could be identified. Low risk could be identified if ƩRQ values fall between 0.01 and 0.1. If ƩRQ values fall between 0.1 and 1.0, the risk is at an intermediate level. If ƩRQ values > 10, the risk is at a high level [25]. The PENC values used in the environmental risk assessment are shown in Table S7. The effluent in this study was discharged into the Yangtze River Estuary generally, and PNEC values for algae and fish were selected. This evaluation is important to quantify the impact of the EDCs on the primary productivity and ecosystem structure in the receiving waterbody.

## 3. Result and Discussion

### 3.1. Behavior of MPs in and through Two Typical WWTPs in Megacity

#### 3.1.1. Contamination Patterns of MPs in the Influent and Source Analysis

The abundances of MPs in the influent from the two WWTPs were different, and a significant seasonal variation was also noted. Specifically, the abundances were 93 items/L in WWTP-A and 163 items/L in WWTP-B in the wet season, while 54 items/L WWTP-A and 140 items/L in WWTP-B in the dry season (Table S8). The observed abundance in this study was comparable to those found in India (42–150 items/L) [26]. Furthermore, the abundance in the wet season was significantly higher than that in the dry season. Similar seasonal variation was found in previous studies [27,28]. As previously reported for ions and solids, increased concentration of MPs in influent during the wet season (July) could be explained by sun irradiation, which enhances water evaporation [29]. The higher temperature in the wet season also leads to more plastic consumption and washing activities [8,30]. More water evaporation, plastic consumption, and washing activities (major sources of fibers) comprehensively increased the MPs abundance in the influent.

**Figure 2:**
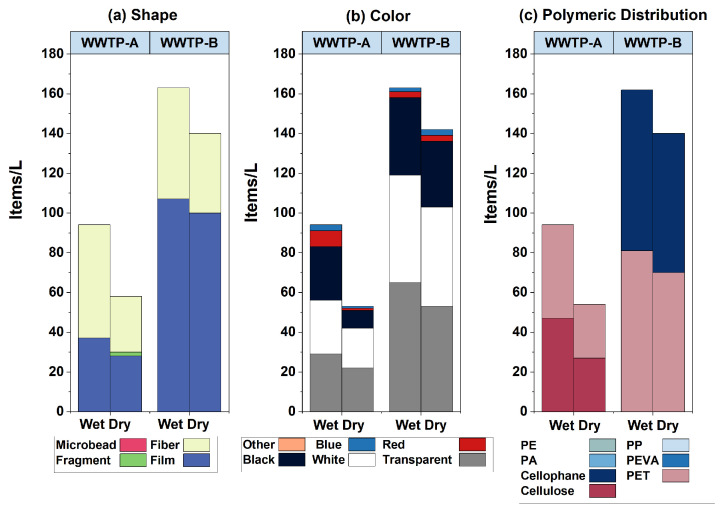
Shape (**a**), color (**b**) and polymeric distribution (**c**) of MPs in the influent samples from the two WWTPs. PE: polyethylene, PP: polypropylene, PA: polyamide, PEVA: polyethylene vinyl acetate, PET: polyethylene glycol terephthalate.

Four sharps of MPs were detected, namely film, fragment, fiber, and microbeads (Figure 2a). In WWTP-A, fiber, which is primarily from domestic washing, was dominated with a ratio of 50–60%. This finding was the same as previous studies [31]. During washing activities, superfine fibers are flushed from the synthetic clothing into the sewage system [32]. As estimated, approximately 100 fibers in 1 L of laundry wastewater enter WWTPs [33]. Insufficient removal of fibers occurs due to their smaller size and lower density [6] and the residues are introduced into the receiving environment finally. The second dominant sharp was film, generally from the plastics used in packaging [34]. In WWTP-B, the sharp film dominated, followed by fiber, indicating a different source of MPs in the influent. The abundance of microbeads that were used as ingredients in toothpaste and personal care products exhibited the lowest level, indicating the positive effect of the prohibition of microbeads in personal care products [35].

Transparent (31–63%) and white (20–47%) were the most common colors, followed by black (11–40%) and other colors (Figure 2b). Transparent and white MPs were primarily from packaging materials, such as bottles and plastic tableware. The color of MPs determines the hazardous potential to the biota. Transparent and white MPs are easily ingested by zooplankton, fish, and other species, causing environmental risks [36]. Therefore, it is crucial to characterize the color of MPs and assess their potential risks.

Polymeric distribution of the MPs was determined. Polyethylene glycol terephthalate (PET) (50%) and cellulose (50%) dominated WWTP-A, while PET (50%) and cellophane (50%) dominated WWTP-B (Figure 2c). No significant seasonal variation in polymeric distribution was observed. PET was the major polymer in all the samples, mainly due to its extensive application in synthetic clothing and food packaging [37]. Cellulose has been used widely in industries such as mechanical engineering and biopharmaceutical [38], indicating a wastewater source from these industries in WWTP-A. Cellophane has been confirmed to be generated from washing activities, suggesting a domestic source [39]. Different polymeric distributions indicate a different water source for the two WWTPs.

#### 3.1.2. Behavior of MPs through the WWTP

The behavior of MPs through the WWTP in different seasons was studied. In general, the abundance of MPs reduced. The removal rates in WWTP-A in the wet and dry seasons were 82.1% and 63.9%, while those in WWTP-B were 92.1% and 88.6% (Table S6). Removal rates in the wet season were higher than those in the dry season.

**Figure 3:**
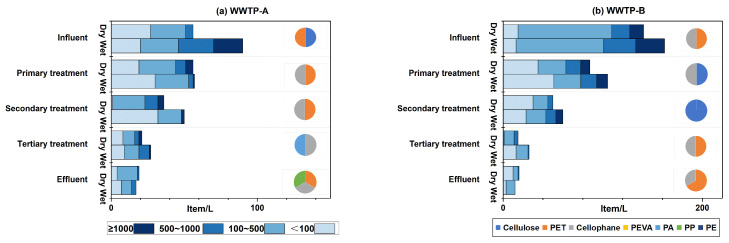
Size and percentage polymeric distribution of MPs in different compartments in the WWTP-A (**a**) and WWTP-B (**b**).

Figure 3 shows the changes in size and polymeric distribution of MPs in different compartments. MP size determined the removal efficiency significantly. Generally, the MPs with sizes >500 μm were reduced significantly from influent to secondary treatment (Table S9) in both WWTPs. However, it is noted that the abundance of MPs with sizes 500–1000 μm and ≥1000 increased with a ratio of 27 (Table S9) in the dry season in WWTP-A. The last step in primary treatment in WWTP-A is a sedimentation tank, which may release the settled or adsorbed MPs back into the water phase [40]. Also, there is a slight increase after the secondary sedimentation of the tertiary treatment, possibly due to the resuspension from the sludge in this process [40]. For those MPs between 100 and 500 μm, the abundance did not change significantly in WWTP-A, while it reduced notably in WWTP-B. For the MPs even smaller (<100 μm), the primary treatment did not perform well, while the tertiary showed better efficiency. Finally, in the effluent, the MPs within the 100–500 μm range were more frequently observed, followed by those smaller than 100 μm. The smaller pieces float more easily, which impedes their settling into the sludge [41].

PET was removed sufficiently for the most abundant polymer. Especially in WWTP-B, PET was removed at a rate of 100% (Table S10). The high reduction of PET in the WWTP was likely due to its high density (1.38 g/cm^3^) [28,42]. However, PET concentration was elevated after the tertiary treatment compartment. The release of PET from sludge or adsorption materials may explain this increase. Furthermore, the polymer polypropylene (PP), which was not detected in the influent in both WWTPs, was observed in the effluent, indicating a considerable release from the PP pipelines used in the WWTPs system [43]. Conclusively, WWTPs performed well for MPs, especially for those with large size and high density.

### 3.2. EDCs in the WWTPs

#### 3.2.1. EDCs in the Influent and Source Diagnosis

**Figure 4:**
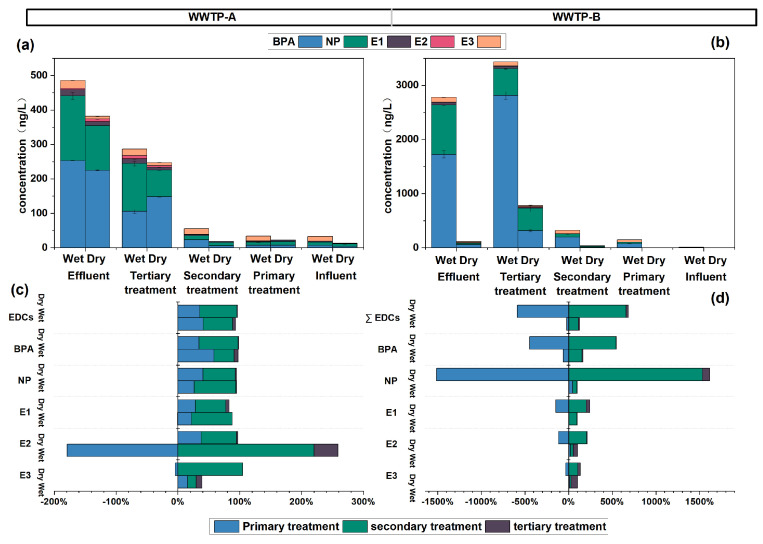
EDCs in different treatment compartments (**a**,**b**) and the removal rates (**c**,**d**).

In this study, seven EDCs were analyzed in the samples collected in March (dry season) as well as in July (wet season) from the WWTPs. The results are shown in Figure 4a, b, and Table S11, where ∑EDCs concentrations in the influent from WWTP-A were found to be 485.46 ± 10.98 ng/L in the wet season and 381.85 ± 0.54 ng/L in the dry season, while those from WWTP-B were 2780.45 ± 59.03 ng/L and 113.85 ± 0.61 ng/L, respectively. Five target EDCs (BPA, NP, E1, E2, and E3) were detected in the influent water from both WWTPs, and the dominant individuals were BPA and NP. BPA is generally from paper mills, fine chemical facilities, and plastics production, while NP comes from the production of detergent products and textile auxiliaries [14,44]. The presence of BPA and NP also suggested that industrial wastewater is a significant influent source of the two WWTPs.

#### 3.2.2. The Removal Efficiencies of EDCs through Different Treatment Compartments

After the treatments, the concentration was reduced significantly (Figure 4a,b, and Table 2). Specifically, the removal rates of total EDCs were 93.22% and 96.52% in the wet and dry seasons in WWTP-A, while 99.64% and 98.98% in WWTP-B, respectively.

In terms of the removal efficiencies in different treatment compartments, the primary treatment in WWTP-A eliminated total EDCs with a rate ranging from 35.3% (dry) to 40.9% (wet), and the secondary process removed EDCs with a percentage of 80.7% (wet)–82.6% (dry) (Table 2). Tertiary treatment, as well as disinfection before discharge, did not significantly remove EDCs. Unlike WWTP-A, primary treatment in WWTP-B elevated EDCs concentration in both studied seasons (Figure 4a,b, Table 2 and Table S11), which may be related to the re-suspended of these EDCs from the sludge. The secondary and tertiary treatments (ozone oxidation included) performed well, with their removal rates higher than 90%. Filtration, sedimentation, and adsorption are basic processes in the primary treatments to remove contaminants; biodegradation and bio-absorption are major processes in the secondary treatments, and in tertiary treatments, the contaminants could be eliminated through advanced oxidation or adsorption processes. In conclusion, it is demonstrated that the principal process for removing EDCs from wastewater is biochemical degradation during secondary treatment. Advanced oxidation is another effective process, as confirmed by WWTP-B.

In addition, there were large differences in the removal efficiency of the five detected EDC individuals. As shown in Figure 4c, in WWTP-A, removal variations of phenolic estrogen in different seasons were not significant, while those of steroid hormones in different seasons were significantly non-negligible. The performance in the wet season is better than in the dry season. As concluded in the last paragraph, the principal processes for removing EDCs from wastewater are biochemical degradation during secondary treatment, and higher temperature leads to enhanced biodegradation [45]. Also, E2 and E3 were released during the primary treatment. This may be due to the reconversion of inactive conjugated steroidal estrogens to active forms, ultimately leading to an increase in their concentrations [46]. In the tertiary treatment, the EDCs were removed but with a low removal efficiency (Table 2). Our result was comparable to previous studies in that micro-activities removed EDCs rather than sedimentation [47].

EDC individuals exhibited different behaviors in WWTP-B. Concentrations in WWTP-B were magnitudes higher in the wet season than those in the dry season. As plotted in Figure 4d, there were significant releases of BPA and NP after the primary treatment. Aeration processes in the primary treatment may lead to a release of BPA and NP from the sludge [45]. E1, E2, and E3 increased as well due to the uncoupling of the sulfate and glucuronide affixes of EDCs during the treatments [48]. This result emphasized that conjugate compounds play a non-negligible role in the removal of EDCs and deserve further exploration. Although there were releases from the primary treatment, the secondary treatment in WWTP-B showed a good removal for all five monomers. The phenolic estrogens (88.03% to 98.85%) were removed more efficiently than steroidal estrogens (18.24% to 96.64%) (Table 2). In summary, although some monomers presented an elevated trend in certain treatment compartments, both wastewater plants can remove more than 80% of the EDCs. The compartment (secondary treatment) based on biodegradation played a more important role.

### 3.3. Relationship between EDCs and MPs

**Figure 5:**
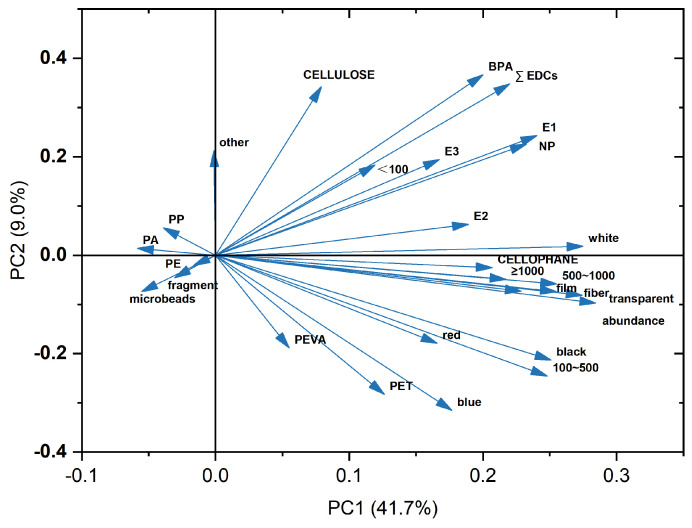
PCA analysis based on MP abundance, their properties, and EDC concentrations.

To further confirm the possibility of MPs and EDCs as each other’s indicators, a principal component analysis (PCA) was applied, and the result is given in Figure 5. As indicated, the concentration of EDCs and MPs with small size (<100 μm) was significantly correlated, indicating a potential of these MPs carrying or releasing the EDCs [49]. In a previous study on the environmental behavior of plastic additives [50], it was demonstrated that the presence of BPA in the water column is highly related to the migration of additives from plastic containers. Additionally, EDC levels negatively correlated to the abundance of PP, PA, and PE. These components of MPs possibly absorbed and accumulated EDCs on their surface due to the homogeneous structure and strong hydrophobicity, finally reducing EDC concentration in the dissolved phase. In summary, the correlation between EDCs and MPs suggested that the size and composition of MPs may affect the concentration of EDCs. More research regarding MPs as a contamination indicator for EDCs should be carried out in the future.

### 3.4. Risk Reduction by the WWTPs

Compared to the safe value (6650 items/m^3^) proposed by Everaert, et al. [22], the abundances in the influent (53,500–162,667 items/m^3^) and effluent (12,833–22,677 items/m^3^) were significantly higher than the safe concentration. Although the MPs were removed efficiently, possible risk still could be initiated by the effluent. Comparatively, the MPs abundances in both influent (171,000 items/m^3^) and effluent (12,800 items/m^3^) from a WWTP from Spain were higher than the safe concentration [51]. Similar results were found in another WWTP in a big city, Xi’an, in China [52]. To evaluate the environmental risk more accurately, the ecological risk index of the WWTP wastewater was determined (Table 3). As given in the table, the wastewater would induce a risk at level II without WWTP treatments. After a series of treatments, the risk index values were reduced to a range of 1.89 to 4.28, which were at an I level. From the influent (RI = 8.1–24.5) to effluent (RI = 1.25–4.28), WWTPs significantly reduced the environmental risk initiated by MPs. It is noted that the risk value was not considered for cellulose and cellophane due to their limited hazardous data. The RI values were only calculated based on the PE, PA, PP, PEVA, and PET, leading to a possible underestimation.

The risk of EDCs was assessed based on the risk quotient method. As given in Table 4, both influent (RQ = 0.106–3.08) and effluent (RQ = 0.0014–0.024) pose limited risk to algae. The reason is that the EDCs primarily target endocrine systems, which algae does not have. After WWTPs, the risk of EDCs for algae reduced from intermediate to low. Algae is an important primary producer in an ecosystem. The removal of the EDCs reduced the environmental pressure on algae and protected the stability of the ecosystem. For fish, the risk of EDCs from WWTPs could not be ignored. Although the risk reduction rates (83.9–98.8%) were high, the residual levels of EDCs still trigger high risk to fish. Specifically, the RQ values of effluent ranged from 3.40 to 30.1, and the risk was primarily induced by E1 and BPA. The receiving water of the two WWTPs is an important waterbody for rare or endangered species, such as Chinese sturgeon and estuarine taper tail anchovy. Similarly, previous studies found environmental threats due to EDCs (BPA, E1, and E2) in the effluent from WWTPs in Europe [53,54].

Thus, technical improvement is needed in WWTPs, especially for EDCs. Advanced oxidation is an effective option for the removal of EDCs. Some studies found that advanced oxidation using ozone could remove more than 90% of the residual EDCs [55,56,57], and WWTPs coupled with wetlands may further reduce the environmental risk of the effluent [58,59,60].

## 4. Conclusions

This study confirmed that the influence of the two WWTPs in the megacity of Shanghai was from both domestic and industrial wastewater. The abundance of MPs was higher in the wet season due to the higher temperature, increased plastic consumption, and washing activities. The removal of MPs in the WWTPs was good, and the wet season performed better than the dry season. MPs of large size and density were more easily removed by sedimentation, while those with small size and density were more difficult to remove. For EDCs, the removal was good as well, ranging from 93.2% to 99.6%. However, the removals of different EDC individuals varied considerably in different seasons and WWTPs. MPs could also be considered an indicator of EDC contamination in the WWTPs. MPs possibly released EDCs during the water treatment processes. Finally, the WWTPs cut off the environmental risk of wastewater significantly and reduced the environmental pressure on the receiving water bodies. The environmental risk caused by MPs in the effluent is low, but EDCs still cause non-negligible negative environmental impacts on fish and ultimately threaten the stability of the ecosystem. Technical improvement is needed in WWTPs, especially for EDCs. Advanced oxidation techniques, such as ozone oxidation, should be applied. WWTP coupled with wetlands is another alternative option. Also, the reuse of effluent water in a particular industry or agriculture instead of directly discharging it should be considered.

## Figures and Tables

**Table 1:** Risk-level criteria for microplastic pollution.

Potential Ecological Risk Factor	Ecological Risk level
<10	I
10–100	II
100–1000	III
>1000	IV

**Table 2:** The removal rates of the EDCs through the two typical WWTPs in different seasons.

		Wet				Dry			
		Primary Treatment	Secondary Treatment	Tertiary Treatment	Total Removal Rate	Primary Treatment	Secondary Treatment	Tertiary Treatment	Total Removal Rate
WWTP-A	BPA	58.25%	77.97%	69.90%	97.23%	34.17%	96.12%	34.40%	98.32%
	NP	26.26%	91.46%	24.10%	95.22%	40.29%	87.84%	22.26%	94.36%
	E1	21.74%	84.41%	−3.31%	87.39%	27.83%	68.44%	24.26%	82.75%
	E2	−178.99%	78.91%	65.90%	79.93%	37.33%	91.01%	44.88%	96.89%
	E3	15.50%	16.47%	13.60%	39.02%	−4.53%	100.00%	100.00%	100.00%
	∑ EDCs	40.91%	80.67%	40.62%	93.22%	35.30%	92.63%	26.92%	96.52%
WWTP-B	BPA	63.25%	92.98%	97.86%	99.76%	−447.56%	98.95%	76.17%	98.63%
	NP	46.13%	88.03%	91.03%	99.42%	−1513.88%	94.80%	99.45%	99.54%
	E1	2.67%	96.44%	64.68%	98.77%	−146.65%	81.77%	90.55%	95.75%
	E2	20.73%	40.23%	100.00%	100.00%	−115.30%	96.64%	100.00%	100.00%
	E3	13.33%	18.24%	100.00%	100.00%	−33.71%	77.80%	100.00%	100.00%
	∑ EDCs	−23.54%	90.57%	96.89%	99.64%	−585.78%	95.71%	96.52%	98.98%

**Table 3:** MPs risk evaluation in the influent and effluent in the WWTPs.

WWTPs	Sampling Season	Influent RI	Risk Level of Influent	Effluent RI	Risk Level of Effluent	Risk Reduction Rate (%)
WWTP-A	Wet	14.0	II	1.89	I	86.51%
	Dry	8.1	II	1.25	I	84.42%
WWTP-B	Wet	24.5	II	3.43	I	85.97%
	Dry	21.1	II	4.28	I	79.68%

**Table 4:** EDCs risk evaluation in the influent and effluent in the WWTPs.

Risk to Algae						
WWTPs	Sampling Season	Influent RQ	Influent Risk Level	Effluent RQ	Effluent Risk Level	Risk Reduction Rate (%)
WWTP-A	Wet	0.547	Intermediate	0.024	Low	95.7%
	Dry	0.428	Intermediate	0.0175	Low	95.9%
WWTP-B	Wet	3.08	High	0.0126	Low	99.6%
	Dry	0.106	Intermediate	0.0014	Low	98.7%
**Risk to fish**						
WWTP-A	Wet	239	High	30.1	high	87.4%
	Dry	160	High	25.8	high	83.9%
WWTP-B	Wet	648	High	7.54	high	98.8%
	Dry	87.8	High	3.40	high	96.1%

## Data Availability

The data presented in this study are available on request from the corresponding author.

## Supplementary Materials

The following supporting information can be downloaded at: https://www.mdpi.com/article/10.3390/toxics12050345/s1, Table S1: List of abbreviations; Table S2: Instrumental parameters for the target compounds; Table S3: Mass spectrum monitoring conditions of target compounds and internal standards; Table S4: Physicochemical properties of the target contaminants; Table S5: The recoveries (%), method detection limits (MDLs), and limits of quantification (LOQs) of the contaminants; Table S6: Ranking of plastic polymers based on hazard classifications of monomers; Table S7: PNECs values used in the environmental risk assessment for EDCs; Table S8: PAbundance of MPs with different sizes in different compartments; Table S9: Removal efficiencies of MPs with different sizes of each treatment compartment; Table S10: Removal rates of different polymers through the WWTPs; Table S11: Concentrations of different EDCs in different compartments. References [61,62,63,64,65] are cited in the supplementary materials

## References

[1] Rodriguez-Mozaz S., Lopez de Alda M.J., Barceló D. (2007). Advantages and limitations of on-line solid phase extraction coupled to liquid chromatography–mass spectrometry technologies versus biosensors for monitoring of emerging contaminants in water. J. Chromatogr. A.

[2] Al Aukidy M., Verlicchi P., Jelic A., Petrovic M., Barcelò D. (2012). Monitoring release of pharmaceutical compounds: Occurrence and environmental risk assessment of two WWTP effluents and their receiving bodies in the Po Valley, Italy. Sci. Total Environ..

[3] Rehman M.U., Nisar B., Mohd Yatoo A., Sehar N., Tomar R., Tariq L., Ali S., Ali A., Mudasir Rashid S., Bilal Ahmad S. (2024). After effects of Pharmaceuticals and Personal Care Products (PPCPs) on the biosphere and their counteractive ways. Sep. Purif. Technol..

[4] Stasinakis A.S., Thomaidis N.S., Arvaniti O.S., Asimakopoulos A.G., Samaras V.G., Ajibola A., Mamais D., Lekkas T.D. (2013). Contribution of primary and secondary treatment on the removal of benzothiazoles, benzotriazoles, endocrine disruptors, pharmaceuticals and perfluorinated compounds in a sewage treatment plant. Sci. Total Environ..

[5] Murphy F., Ewins C., Carbonnier F., Quinn B. (2016). Wastewater Treatment Works (WwTW) as a Source of Microplastics in the Aquatic Environment. Environ. Sci. Technol..

[6] Talvitie J., Mikola A., Koistinen A., Setälä O. (2017). Solutions to microplastic pollution—Removal of microplastics from wastewater effluent with advanced wastewater treatment technologies. Water Res..

[7] Ren S.-Y., Sun Q., Xia S.-Y., Tong D., Ni H.-G. (2023). Microplastics in wastewater treatment plants and their contributions to surface water and farmland pollution in China. Chemosphere.

[8] Ziajahromi S., Neale P.A., Leusch F.D.L. (2016). Wastewater treatment plant effluent as a source of microplastics: Review of the fate, chemical interactions and potential risks to aquatic organisms. Water Sci. Technol..

[9] Sol D., Laca A., Laca A., Díaz M. (2020). Approaching the environmental problem of microplastics: Importance of WWTP treatments. Sci. Total Environ..

[10] Mato Y., Isobe T., Takada H., Kanehiro H., Ohtake C., Kaminuma T. (2001). Plastic Resin Pellets as a Transport Medium for Toxic Chemicals in the Marine Environment. Environ. Sci. Technol..

[11] Sun P., Liu X., Zhang M., Li Z., Cao C., Shi H., Yang Y., Zhao Y. (2021). Sorption and leaching behaviors between aged MPs and BPA in water: The role of BPA binding modes within plastic matrix. Water Res..

[12] Wee S.Y., Aris A.Z. (2017). Endocrine disrupting compounds in drinking water supply system and human health risk implication. Environ. Int..

[13] Kumar P., Shimali, Chamoli S., Khondakar K.R. (2023). Advances in optical and electrochemical sensing of bisphenol a (BPA) utilizing microfluidic Technology: A mini perspective. Methods.

[14] Devi T., Saleh N.M., Kamarudin N.H.N., Roslan N.J., Jalil R., Hamid H.A. (2023). Efficient adsorption of organic pollutants phthalates and bisphenol A (BPA) utilizing magnetite functionalized covalent organic frameworks (MCOFs): A promising future material for industrial applications. Ecotoxicol. Environ. Saf..

[15] Shi W.-J., Jiang Y.-X., Huang G.-Y., Zhao J.-L., Zhang J.-N., Liu Y.-S., Xie L.-T., Ying G.-G. (2018). Dydrogesterone Causes Male Bias and Accelerates Sperm Maturation in Zebrafish (Danio rerio). Environ. Sci. Technol..

[16] Xu E.G.B., Liu S., Ying G.-G., Zheng G.J.S., Lee J.H.W., Leung K.M.Y. (2014). The occurrence and ecological risks of endocrine disrupting chemicals in sewage effluents from three different sewage treatment plants, and in natural seawater from a marine reserve of Hong Kong. Mar. Pollut. Bull..

[17] Stasinakis A.S., Gatidou G., Mamais D., Thomaidis N.S., Lekkas T.D. (2008). Occurrence and fate of endocrine disrupters in Greek sewage treatment plants. Water Res..

[18] Su L., Xue Y., Li L., Yang D., Kolandhasamy P., Li D., Shi H. (2016). Microplastics in Taihu Lake, China. Environ. Pollut..

[19] Su L., Nan B., Craig N.J., Pettigrove V. (2020). Temporal and spatial variations of microplastics in roadside dust from rural and urban Victoria, Australia: Implications for diffuse pollution. Chemosphere.

[20] Hakanson L. (1980). An ecological risk index for aquatic pollution control.a sedimentological approach. Water Res..

[21] Peng G., Xu P., Zhu B., Bai M., Li D. (2018). Microplastics in freshwater river sediments in Shanghai, China: A case study of risk assessment in mega-cities. Environ. Pollut..

[22] Everaert G., Van Cauwenberghe L., De Rijcke M., Koelmans A.A., Mees J., Vandegehuchte M., Janssen C.R. (2018). Risk assessment of microplastics in the ocean: Modelling approach and first conclusions. Environ. Pollut..

[23] Lithner D., Larsson Å., Dave G. (2011). Environmental and health hazard ranking and assessment of plastic polymers based on chemical composition. Sci. Total Environ..

[24] Sharma B.M., Bečanová J., Scheringer M., Sharma A., Bharat G.K., Whitehead P.G., Klánová J., Nizzetto L. (2019). Health and ecological risk assessment of emerging contaminants (pharmaceuticals, personal care products, and artificial sweeteners) in surface and groundwater (drinking water) in the Ganges River Basin, India. Sci. Total Environ..

[25] Hernando M.D., Mezcua M., Fernández-Alba A.R., Barceló D. (2006). Environmental risk assessment of pharmaceutical residues in wastewater effluents, surface waters and sediments. Talanta.

[26] Parashar N., Hait S. (2023). Abundance, characterization, and removal of microplastics in different technology-based sewage treatment plants discharging into the middle stretch of the Ganga River, India. Sci. Total Environ..

[27] Bayo J., Olmos S., López-Castellanos J. (2020). Microplastics in an urban wastewater treatment plant: The influence of physicochemical parameters and environmental factors. Chemosphere.

[28] Long Z., Pan Z., Wang W., Ren J., Yu X., Lin L., Lin H., Chen H., Jin X. (2019). Microplastic abundance, characteristics, and removal in wastewater treatment plants in a coastal city of China. Water Res..

[29] Bayo J., López-Castellanos J. (2016). Principal factor and hierarchical cluster analyses for the performance assessment of an urban wastewater treatment plant in the Southeast of Spain. Chemosphere.

[30] Carr S.A., Liu J., Tesoro A.G. (2016). Transport and fate of microplastic particles in wastewater treatment plants. Water Res..

[31] Gies E.A., LeNoble J.L., Noël M., Etemadifar A., Bishay F., Hall E.R., Ross P.S. (2018). Retention of microplastics in a major secondary wastewater treatment plant in Vancouver, Canada. Mar. Pollut. Bull..

[32] Yang T., Gao M., Nowack B. (2023). Formation of microplastic fibers and fibrils during abrasion of a representative set of 12 polyester textiles. Sci. Total Environ..

[33] Browne M.A., Crump P., Niven S.J., Teuten E., Tonkin A., Galloway T., Thompson R. (2011). Accumulation of Microplastic on Shorelines Woldwide: Sources and Sinks. Environ. Sci. Technol..

[34] Yin L., Wen X., Du C., Jiang J., Wu L., Zhang Y., Hu Z., Hu S., Feng Z., Zhou Z. (2020). Comparison of the abundance of microplastics between rural and urban areas: A case study from East Dongting Lake. Chemosphere.

[35] Lin W.-H., Ou J.-H., Yu Y.-L., Liu P.-F., Surampalli R.Y., Kao C.-M. (2023). Regulatory Framework of Microconstituents. Microconstituents in the Environment.

[36] Tavelli R., Callens M., Grootaert C., Abdallah M.F., Rajkovic A. (2022). Foodborne pathogens in the plastisphere: Can microplastics in the food chain threaten microbial food safety?. Trends Food Sci. Technol..

[37] Wang W., Ndungu A.W., Li Z., Wang J. (2017). Microplastics pollution in inland freshwaters of China: A case study in urban surface waters of Wuhan, China. Sci. Total Environ..

[38] Ragi K.B., Ekka B., Mezule L. (2022). Zero pollution protocol for the recovery of cellulose from municipal sewage sludge. Bioresour. Technol. Rep..

[39] Harley-Nyang D., Memon F.A., Jones N., Galloway T. (2022). Investigation and analysis of microplastics in sewage sludge and biosolids: A case study from one wastewater treatment works in the UK. Sci. Total Environ..

[40] Long Y., Zhou Z., Yin L., Wen X., Xiao R., Du L., Zhu L., Liu R., Xu Q., Li H. (2022). Microplastics removal and characteristics of constructed wetlands WWTPs in rural area of Changsha, China: A different situation from urban WWTPs. Sci. Total Environ..

[41] Xu Z., Zhai X., Bai X. (2023). Amplifiers of environmental risk of microplastics in sewage sludge: Thermal drying treatment. Sci. Total Environ..

[42] San José R., Pérez J.L., Callén M.S., López J.M., Mastral A. (2013). BaP (PAH) air quality modelling exercise over Zaragoza (Spain) using an adapted version of WRF-CMAQ model. Environ. Pollut..

[43] Tong H., Jiang Q., Hu X., Zhong X. (2020). Occurrence and identification of microplastics in tap water from China. Chemosphere.

[44] Halder A.K., Moura A.S., Cordeiro M.N.D.S. (2023). Predicting the ecotoxicity of endocrine disruptive chemicals: Multitasking in silico approaches towards global models. Sci. Total Environ..

[45] Brachi P., Di Fraia S., Massarotti N., Vanoli L. (2022). Combined heat and power production based on sewage sludge gasification: An energy-efficient solution for wastewater treatment plants. Energy Convers. Manag. X.

[46] Yu Q., Yang X., Zhao F., Hu X., Guan L., Ren H., Geng J. (2022). Spatiotemporal variation and removal of selected endocrine-disrupting chemicals in wastewater treatment plants across China: Treatment process comparison. Sci. Total Environ..

[47] Sun J., Wang J., Zhang R., Wei D., Long Q., Huang Y., Xie X., Li A. (2017). Comparison of different advanced treatment processes in removing endocrine disruption effects from municipal wastewater secondary effluent. Chemosphere.

[48] Ben W., Zhu B., Yuan X., Zhang Y., Yang M., Qiang Z. (2017). Transformation and fate of natural estrogens and their conjugates in wastewater treatment plants: Influence of operational parameters and removal pathways. Water Res..

[49] Mo Q., Yang X., Wang J., Xu H., Li W., Fan Q., Gao S., Yang W., Gao C., Liao D. (2021). Adsorption mechanism of two pesticides on polyethylene and polypropylene microplastics: DFT calculations and particle size effects. Environ. Pollut..

[50] Guart A., Bono-Blay F., Borrell A., Lacorte S. (2011). Migration of plasticizersphthalates, bisphenol A and alkylphenols from plastic containers and evaluation of risk. Food Addit. Contam. Part A.

[51] Edo C., González-Pleiter M., Leganés F., Fernández-Piñas F., Rosal R. (2020). Fate of microplastics in wastewater treatment plants and their environmental dispersion with effluent and sludge. Environ. Pollut..

[52] Yang Z., Li S., Ma S., Liu P., Peng D., Ouyang Z., Guo X. (2021). Characteristics and removal efficiency of microplastics in sewage treatment plant of Xi’an City, northwest China. Sci. Total Environ..

[53] Čelić M., Škrbić B.D., Insa S., Živančev J., Gros M., Petrović M. (2020). Occurrence and assessment of environmental risks of endocrine disrupting compounds in drinking, surface and wastewaters in Serbia. Environ. Pollut..

[54] dos Santos D.M., Buruaem L., Gonçalves R.M., Williams M., Abessa D.M.S., Kookana R., de Marchi M.R.R. (2018). Multiresidue determination and predicted risk assessment of contaminants of emerging concern in marine sediments from the vicinities of submarine sewage outfalls. Mar. Pollut. Bull..

[55] Yazdan M.M.S., Kumar R., Leung S.W. (2022). The Environmental and Health Impacts of Steroids and Hormones in Wastewater Effluent, as Well as Existing Removal Technologies: A Review. Ecologies.

[56] Le-Minh N., Khan S.J., Drewes J.E., Stuetz R.M. (2010). Fate of antibiotics during municipal water recycling treatment processes. Water Res..

[57] Almazrouei B., Islayem D., Alskafi F., Catacutan M.K., Amna R., Nasrat S., Sizirici B., Yildiz I. (2023). Steroid hormones in wastewater: Sources, treatments, environmental risks, and regulations. Emerg. Contam..

[58] Fernandes J.P., Almeida C.M.R., Pereira A.C., Ribeiro I.L., Reis I., Carvalho P., Basto M.C.P., Mucha A.P. (2015). Microbial community dynamics associated with veterinary antibiotics removal in constructed wetlands microcosms. Bioresour. Technol..

[59] Song H.-L., Zhang S., Guo J., Yang Y.-L., Zhang L.-M., Li H., Yang X.-L., Liu X. (2018). Vertical up-flow constructed wetlands exhibited efficient antibiotic removal but induced antibiotic resistance genes in effluent. Chemosphere.

[60] Ilyas H., van Hullebusch E.D. (2020). A review on the occurrence, fate and removal of steroidal hormones during treatment with different types of constructed wetlands. J. Environ. Chem. Eng..

[61] Lee C.-C., Jiang L.-Y., Kuo Y.-L., Chen C.-Y., Hsieh C.-Y., Hung C.-F., Tien C.-J. (2015). Characteristics of nonylphenol and bisphenol A accumulation by fish and implications for ecological and human health. Sci. Total Environ..

[62] Wright-Walters M., Volz C., Talbott E., Davis D. (2011). An updated weight of evidence approach to the aquatic hazard assessment of Bisphenol A and the derivation a new predicted no effect concentration (Pnec) using a non-parametric methodology. Sci. Total Environ..

[63] iang R., Liu J., Huang B., Wang X., Luan T., Yuan K. (2020). Assessment of the potential ecological risk of residual endocrine-disrupting chemicals from wastewater treatment plants. Sci. Total Environ..

[64] Lu S., Lin C., Lei K., Xin M., Wang B., Ouyang W., Liu X., He M. (2021). Endocrine-disrupting chemicals in a typical urbanized bay of Yellow Sea, China: Distribution, risk assessment, and identification of priority pollutants. Environ. Pollut..

[65] Czarny K., Szczukocki D., Krawczyk B., Skrzypek S., Zieliński M., Gadzała-Kopciuch R. (2019). Toxic effects of single animal hormones and their mixtures on the growth of Chlorella vulgaris and Scenedesmus armatus. Chemosphere.

