# Supplementary material for: Microplastics and Endocrine Disruptors in Typical Wastewater Treatment Plants in Megacity Shanghai
Source: Toxics. 2024 May 8;12(5):345. doi: 10.3390/toxics12050345 (PMC11126012; doi:10.3390/toxics12050345)
Supplement: Supplementary file 1 [file toxics-12-00345-s001.zip › toxics-2965517-supplementary.pdf]

Microplastics and Endocrine Disruptors in Typical Wastewater Treatment Plants in Megacity Shanghai

Yuxiao Tong [1], Manjun Xie [2], Hanwen Xv [1], Ruihua Sun [3], Qian Wang [1,*] and Juan-ying Li [1]

Table S1 List of abbreviations

Compound	Acronym
Wastewater Treatment Plants	WWTP
Microplastics	MPs
Endocrine Disrupting Chemicals	EDCs
Phenolic Estrogens	PEs
Estrogens	SEs
Current-use Pesticides	CUPs
Pharmaceuticals and Personal Care Products	PPCPs
Predicted No-observable Concentration	PNEC
Multiple Reaction Monitoring	MRM
Measured Concentration	MEC
Risk Quotient	RQ
Bisphenol A	BPA
Nonylphenol	NP
Estrone	E1
17β-Estradiol	E2
17α-Ethynylestradiol	EE2
Estriol	E3
Diethylstilbestrol	DES
Polyethylene	PE
Polypropylene	PP
Polyamide	PA
Polyethylene vinyl acetate	PEVA
Polyethylene glycol terephthalate.	PET

Table S2 Instrumental parameters for the target compounds

<table>
<tr><td rowspan="15">EDCs</td><td colspan="2">Ionization mode</td><td colspan="8">Positive/negative ionization</td></tr>
<tr><td rowspan="7">Liquid chromatography instrument conditions</td><td rowspan="2">Flow phase</td><td>A</td><td colspan="7">0.1% Ammonia solution</td></tr>
<tr><td>B</td><td colspan="7">Acetitrile</td></tr>
<tr><td rowspan="3">Elusion gradient</td><td>Time (min)</td><td>0</td><td>0.5</td><td>2</td><td>12</td><td>13</td><td>13.1</td><td>15</td></tr>
<tr><td>A (%)</td><td>100</td><td>50</td><td>50</td><td>0</td><td>0</td><td>100</td><td>100</td></tr>
<tr><td>B (%)</td><td>0</td><td>50</td><td>50</td><td>100</td><td>100</td><td>0</td><td>0</td></tr>
<tr><td>Flow rate</td><td colspan="8">0.3 mL/min</td></tr>
<tr><td>Column temperature</td><td colspan="8">40 °C</td></tr>
<tr><td rowspan="7">Mass spectrometry instrument conditions</td><td>Dry gas temperature</td><td colspan="8">300 °C</td></tr>
<tr><td>Dry air flow rate</td><td colspan="8">7 L/min</td></tr>
<tr><td>Atomizer pressure</td><td colspan="8">3.10 × 105 Pa (45 psi)</td></tr>
<tr><td>Sheath air flow rate</td><td colspan="8">11 L/min</td></tr>
<tr><td>Sheath gas temperature</td><td colspan="8">350 °C</td></tr>
<tr><td>Capillary voltage</td><td colspan="8">3500 V</td></tr>
<tr><td>Nozzle voltage</td><td colspan="8">500 V</td></tr>
</table>

Table S3 Mass spectrum monitoring conditions of target compounds and internal standards

contaminant	classes	Compound	retention time (min)	CRID	Precursor (m/z)	Product (m/z)	Fragmentor (V)	Collision Pressure (V)
EDCs	PEs	BPA	3.2	BPA-d14	287.2	144.9*	115	40
						170.9		32
		NP	9.7	BPA-d14	219.3	132.9*	95	36
						147		28
	SEs	E1	4.3	BPA-d14	269.2	145*	150	40
						183		40
		E2	3.5	BPA-d14	271.2	145*	100	40
						183		44
		EE2	4.0	BPA-d14	295.3	145*	45	44
						159		36
		E3	1.9	BPA-d14	287.2	144.9*	115	40
						170.9		32
	N-SEs	DES	6.1	BPA-d14	267.2	236.9*	120	28
						251.2		20
	Internal Standard Substance	BPA-d14	3.1	/	241.2	223*	45	16
						142.3		24

Note: Those with * are fixed quantum ions of the secondary mass spectrum

Table S4 Physicochemical properties of the target contaminants

Category	Compound	Acronym	CAS No.	Formula	MW	pKa	logKow
Phenolic estrogenic compounds (PEs)	Bisphenol A	BPA	80-5-7	$C_{15}H_{16}O_2$	228.3	10.2	3.81
Phenolic estrogenic compounds (PEs)	Nonylphenol	NP	25154-52-3	$C_{15}H_{24}O$	220.3	9.8	5.66
Steroid estrogens (SEs)	Estrone	E1	53-16-7	$C_{18}H_{22}O_2$	270.4	10.3	4.03
Steroid estrogens (SEs)	17β-Estradiol	E2	50-28-2	$C_{18}H_{24}O_2$	272.4	10.3	3.57
Steroid estrogens (SEs)	17α-Ethynylestradiol	EE2	57-63-6	$C_{20}H_{24}O_2$	296.4	10.3	3.63
Steroid estrogens (SEs)	Estriol	E3	50-27-1	$C_{18}H_{24}O_3$	288.4	10.3	2.54
Non-steroid estrogens (N-SEs)	Diethylstilbestrol	DES	56-53-1	$C_{18}H_{20}O_2$	268.15		5.64

Table S5 The recoveries (%), method detection limits (MDLs), and limits of quantification (LOQs) of the contaminants

Contaminants	Internal Standard	R^2	Recovery (%)	MDLs (μg/L)	LOQs (ng/L)
BPA	BPA-d14	0.9993	99.2	0.075	0.023
NP	BPA-d14	0.9996	85.6	0.369	0.111
E1	BPA-d14	0.9992	90.8	0.814	0.244
E2	BPA-d14	0.9988	88.5	0.476	0.143
EE2	BPA-d14	0.9993	86.2	0.933	0.280
E3	BPA-d14	0.9968	94.0	0.667	0.200
DES	BPA-d14	0.9994	96.9	0.933	0.280

Table S6 Ranking of plastic polymers based on hazard classifications of monomers.

Polymer	Hazard score	Monomer 1 (wt.%)
Polypropylene (PP)	1 (I)	Propylene (100 wt.%)<br>Flam. Gas 1 (I)
Polyethylene terephthalate (PET)	4 (II)	Ethylene glycol (39 wt.%)<br>Acute Tox. 4[o] (II)
Ethylene vinyl acetate (PEVA)	9 (II)	Ethylene (80 wt.%)<br>STOT SE 3[dd] (II), Flam. Gas. 1 (I)
Polyacrylic acid (PA)	230 (III)	Acrylic acid (100 wt.%)<br>Aq. Acute 1 (III), Skin Corr. 1A (III), Acute Tox. 4[o,d,i] (III)

o,d,i: Toxic by oral, dermal and inhalation route, applies to Acute Tox. categories.

dd: May cause drowsiness or dizziness; rimay cause respiratory irritation, applies to STOT SE 3 classifications.

wt.%: percentage by weight.

Table S7 PNECs values used in the environmental risk assessment for EDCs

Contaminants	fish PNEC (ng/L)	References	Algae PNEC (ng/L)	References
BPA	60	[1]	1000	[2]
NP	330	[1]	721	[3]
E1	0.078	[4]	570	[4]
E2	1	[4]	2480	[4]
E3	46.2	[4]	208390	[5]

Table S8 Abundance of MPs with different sizes in different compartments

Wet						
Abundance (items/L)		<100	100~500	500~1000	≥1000	$\sum MPs$
WWTP-A	Influent	20	26	24	20	93
	Primary treatment	30	23	3	1	57
	Secondary treatment	32	16	0	2	50
	Tertiary treatment	9	10	7	1	27
	Effluent	7	7	3	0	17
WWTP-B	Influent	13	88	32	29	163
	Primary treatment	51	27	16	11	105
	Secondary treatment	23	20	10	7	60
	Tertiary treatment	13	12	1	0	26
	Effluent	3	9	0	0	13
Dry						
WWTP-A	Influent	27	24	5	0	54
	Primary treatment	19	25	7	5	55
	Secondary treatment	1	22	9	4	37
	Tertiary treatment	8	8	3	2	21
	Effluent	4	14	1	0	19
WWTP-B	Influent	15	94	18	14	140
	Primary treatment	35	28	15	9	87
	Secondary treatment	30	15	5	0	50
	Tertiary treatment	1	10	4	0	16
	Effluent	10	5	1	0	16

Table S9 Removal efficiencies of MPs with different sizes of each treatment compartment

Removal rate (%)		Primary treatment	Secondary treatment	Tertiary treatment	Total removal rate
Wet					
WWTP-A	< 100	-46.51%	-8.95%	78.62%	65.87%
	100~500	12.82%	28.52%	57.79%	73.69%
	500~1000	85.74%	95.06%	-1640.00%	87.75%
	⩾1000	96.17%	-95.97%	100.00%	100.00%
	$\sum MPs$	39.46%	11.50%	66.67%	82.14%
WWTP-B	< 100	-284.58%	54.50%	85.48%	74.59%
	100~500	68.94%	27.44%	56.44%	90.18%
	500~1000	50.52%	37.89%	94.95%	98.45%
	⩾1000	64.02%	38.42%	95.23%	98.94%
	$\sum MPs$	35.45%	43.33%	78.43%	92.11%
Dry					
WWTP-A	< 100	31.78%	92.25%	-207.81%	83.73%
	100~500	-2.19%	11.87%	37.77%	43.96%
	500~1000	-37.50%	-27.48%	85.51%	74.61%
	⩾1000	-2700.00%	15.01%	100.00%	100.00%
	$\sum MPs$	-3.43%	34.04%	47.03%	63.86%
WWTP-B	< 100	-143.19%	14.93%	66.67%	31.03%
	100~500	70.56%	45.51%	66.67%	94.65%
	500~1000	18.01%	66.12%	86.67%	96.30%
	⩾1000	32.50%	98.24%	-100.00%	97.62%
	$\sum MPs$	37.86%	42.53%	68.00%	88.57%

Table S10 Removal rates of different polymers through the WWTPs

Removal rate (%)	Polymetric component	Primary treatment	Secondary treatment	Tertiary treatment	Total removal rate
Wet					
WWTP-A	Cellulose	100.00%	0.00%	0.00%	99.46%
	PET	21.43%	31.82%	69.78%	83.81%
	Cellophane	-100.00%	31.82%	69.78%	-100.00%
	PEVA	0.00%	0.00%	0.00%	0.00%
	PA	0.00%	0.00%	0.00%	0.00%
	PP	0.00%	0.00%	-100.00%	-100.00%
	PE	0.00%	0.00%	0.00%	0.00%
WWTP-B	Cellulose	-100.00%	-13.33%	99.93%	0.00%
	PET	99.95%	0.00%	-100.00%	89.48%
	Cellophane	35.45%	99.92%	-100.00%	94.74%
	PEVA	0.00%	0.00%	0.00%	0.00%
	PA	0.00%	0.00%	0.00%	0.00%
	PP	0.00%	0.00%	0.00%	0.00%
	PE	0.00%	0.00%	0.00%	0.00%
Dry					
WWTP-A	Cellulose	99.84%	0.00%	0.00%	99.84%
	PET	-3.43%	34.04%	72.60%	81.31%
	Cellophane	-100.00%	34.04%	72.60%	-100.00%
	PEVA	0.00%	0.00%	0.00%	0.00%
	PA	0.00%	0.00%	0.00%	0.00%
	PP	0.00%	0.00%	-100.00%	-100.00%
	PE	0.00%	0.00%	0.00%	0.00%
WWTP-B	Cellulose	-100.00%	-14.94%	99.92%	0.00%
	PET	99.94%	0.00%	-100.00%	84.76%
	Cellophane	37.86%	99.90%	-100.00%	92.38%
	PEVA	0.00%	0.00%	0.00%	0.00%
	PA	0.00%	0.00%	0.00%	0.00%
	PP	0.00%	0.00%	0.00%	0.00%
	PE	0.00%	0.00%	0.00%	0.00%

Table S11 Concentrations of different EDCs in different compartments

Concentration (ng/L)	Classify	Chemicals	Influent	Primary treatment	Secondary treatment	Effluent	Total removal rate
Wet							
WWTP-A	PEs	BPA	253.34	105.77	23.3	7.42	97.07%
		NP	187.92	138.57	11.84	8.2	95.64%
	$\sum PEs$		441.26	244.34	35.14	15.62	96.46%
	SEs	E1	17.98	14.07	2.19	2.2	87.76%
		E2	3.24	9.03	1.9	2	38.27%
		E3	22.98	19.42	16.22	13.96	39.25%
	$\sum SEs$		44.2	42.52	20.31	18.16	58.91%
	$\sum EDCs$		485.46	286.86	55.45	33.78	93.04%
WWTP-B	PEs	BPA	1724.22	2814.74	197.57	75.63	95.61%
		NP	913.67	492.16	58.93	24.26	97.34%
	$\sum PEs$		2637.89	3306.9	256.5	99.89	96.21%
	SEs	E1	47.46	46.19	1.65	0.75	98.42%
		E2	6.27	4.97	2.97	0.07	98.88%
		E3	88.83	76.99	62.95	48.75	45.12%
	$\sum SEs$		142.56	128.15	67.57	49.57	65.23%
	$\sum EDCs$		2780.45	3435.06	324.08	149.39	94.62%
Dry							
WWTP-A	PEs	BPA	224.91	148.07	5.74	7.47	96.68%
		NP	129.63	77.40	9.41	10.41	91.97%
	$\sum PEs$		129.63	77.4	9.41	10.41	94.96%
	SEs	E1	11.50	8.30	2.62	3.92	65.91%
		E2	7.71	4.83	0.43	0.42	94.55%
		E3	8.09	8.46	0.10	0.10	98.76%
	$\sum SEs$		27.3	21.59	3.15	4.44	83.74%
	$\sum EDCs$		381.85	247.06	18.21	22.22	94.15%
WWTP-B	PEs	BPA	57.12	312.75	3.29	0.79	98.62%
		NP	25.66	414.07	21.52	1.29	94.97%
	$\sum PEs$		82.78	726.82	24.81	2.08	97.49%
	SEs	E1	6.22	15.34	2.80	0.30	95.18%
		E2	6.61	14.22	0.48	0.07	98.94%
		E3	18.25	24.41	5.42	0.49	97.32%
	$\sum SEs$		31.08	53.97	8.7	0.86	97.23%
	$\sum EDCs$		113.86	780.79	33.51	2.94	97.42%

References

1. Lee, C.-C.; Jiang, L.-Y.; Kuo, Y.-L.; Chen, C.-Y.; Hsieh, C.-Y.; Hung, C.-F.; Tien, C.-J. Characteristics of nonylphenol and bisphenol A accumulation by fish and implications for ecological and human health. *Science of The Total Environment* **2015**, *502*, 417-425, doi:https://doi.org/10.1016/j.scitotenv.2014.09.042.
2. Wright-Walters, M.; Volz, C.; Talbott, E.; Davis, D. An updated weight of evidence approach to the aquatic hazard assessment of Bisphenol A and the derivation a new predicted no effect concentration (Pnec) using a non-parametric methodology. *Science of The Total Environment* **2011**, *409*, 676-685, doi:https://doi.org/10.1016/j.scitotenv.2010.07.092.
3. Jiang, R.; Liu, J.; Huang, B.; Wang, X.; Luan, T.; Yuan, K. Assessment of the potential ecological risk of residual endocrine-disrupting chemicals from wastewater treatment plants. *Science of The Total Environment* **2020**, *714*, 136689, doi:https://doi.org/10.1016/j.scitotenv.2020.136689.
4. Lu, S.; Lin, C.; Lei, K.; Xin, M.; Wang, B.; Ouyang, W.; Liu, X.; He, M. Endocrine-disrupting chemicals in a typical urbanized bay of Yellow Sea, China: Distribution, risk assessment, and identification of priority pollutants. *Environmental Pollution* **2021**, *287*, 117588, doi:https://doi.org/10.1016/j.envpol.2021.117588.
5. Czarny, K.; Szczukocki, D.; Krawczyk, B.; Skrzypek, S.; Zieliński, M.; Gadzała-Kopciuch, R. Toxic effects of single animal hormones and their mixtures on the growth of Chlorella vulgaris and Scenedesmus armatus. *Chemosphere* **2019**, *224*, 93-102, doi:https://doi.org/10.1016/j.chemosphere.2019.02.072.
